# Directing visual attention during action observation modulates corticospinal excitability

**DOI:** 10.1371/journal.pone.0190165

**Published:** 2018-01-05

**Authors:** David J. Wright, Greg Wood, Zoe C. Franklin, Ben Marshall, Martin Riach, Paul S. Holmes

**Affiliations:** Motor Cognition Research Group, Research Centre for Health, Exercise and Active Living, Manchester Metropolitan University, Manchester, United Kingdom; University of Bologna, ITALY

## Abstract

Transcranial magnetic stimulation (TMS) research has shown that corticospinal excitability is facilitated during the observation of human movement. However, the relationship between corticospinal excitability and participants’ visual attention during action observation is rarely considered. Nineteen participants took part in four conditions: (i) a static hand condition, involving observation of a right hand holding a ball between the thumb and index finger; (ii) a free observation condition, involving observation of the ball being pinched between thumb and index finger; and (iii and iv) finger-focused and ball-focused conditions, involving observation of the same ball pinch action with instructions to focus visual attention on either the index finger or the ball. Single-pulse TMS was delivered to the left motor cortex and motor evoked potentials (MEPs) were recorded from the first dorsal interosseous (FDI) and abductor digiti minimi muscles of the right hand. Eye movements were recorded simultaneously throughout each condition. The ball-focused condition produced MEPs of significantly larger amplitude in the FDI muscle, compared to the free observation or static hand conditions. Furthermore, regression analysis indicated that the number of fixations on the ball was a significant predictor of MEP amplitude in the ball-focused condition. These results have important implications for the design and delivery of action observation interventions in motor (re)learning settings. Specifically, providing viewing instructions that direct participants to focus visual attention on task-relevant objects affected by the observed movement promotes activity in the motor system in a more optimal manner than free observation or no instructions.

## Introduction

It is well-established that action observation elicits activity in various motor regions of the brain [1, 2, 3]. Furthermore, the manipulation of attention during action observation can modulate activity within the motor system. For example, research using electroencephalography [4, 5] and magnetoencephalography [6] has reported that activity in mu and beta frequency bands over sensorimotor regions of the cortex are increased compared to passive observation when attention is directed towards the observed action using instructions, cognitive tasks, or visual cues. This effect is eliminated when distractor tasks are used to divert attention away from the observed action [7]. Behavioural evidence also indicates that action imitation effects are facilitated when attention is directed towards the observed action [8, 9, 10]. Based on these findings, directing attention explicitly towards the observed action should be an important consideration when delivering action observation interventions for motor (re)learning (see [11]).

Transcranial magnetic stimulation (TMS) is one method that has been used extensively to explore corticospinal excitability during action observation. Fadiga et al. [12] first demonstrated that corticospinal excitability was facilitated during the observation of upper limb movements compared to the observation of static objects. This effect, which was specific to the muscles that would be involved in the execution of the observed action, has since been replicated widely (see [13, 14]). Despite considerable research on this topic, until recently few studies had examined the relationship between visual attention and the facilitation of corticospinal excitability during action observation [15, 16, 17, 18, 19]. Leonetti et al. [15] were among the first to demonstrate that visual attention modulated corticospinal excitability during action observation. They examined differences in the manner in which corticospinal excitability was facilitated when grasping actions were observed in either central or peripheral vision. They reported that passive observation of grasping actions viewed in peripheral vision facilitated corticospinal excitability. This facilitation, however, did not reflect the specific temporal and kinematic components of the observed movement in the manner evident when the same actions were viewed in central vision (e.g., [20]). The authors concluded that when actions are viewed in peripheral vision, the observer may only be able to extract information about general aspects of the observed movement, rather than specific information related to movement execution.

In a related experiment, Donaldson et al. [16] examined the relationship between corticospinal excitability and the location of gaze fixations during passive observation of activities of daily living (e.g., reaching and grasping objects, stirring a cup of tea with a spoon). This study represented an important advancement in the TMS action observation literature as the novel inclusion of eye gaze metrics (e.g., the number or location of fixations) can provide an indication of the attentional processes involved in action observation [21, 22]. Although TMS and eye-tracking measures were not recorded simultaneously, Donaldson et al. reported that facilitation of corticospinal excitability was associated positively with the number of fixations in areas of the video depicting effector-object interactions, and negatively with the number of fixations in areas of the video containing only static objects. The authors interpreted this to reflect greater activation of the putative human mirror neuron system when attending to biological motion.

Taken together, these findings indicate that corticospinal excitability during action observation may be modulated by participants’ visual attention. Specifically, corticospinal excitability may be facilitated to a greater extent when participants attend to task-relevant features of an observed action in central vision. There is evidence that the viewing instructions provided to participants prior to action observation can modulate corticospinal excitability [23, 24, 25]. Instructing participants to fixate on specific task-relevant features of an observed action may, therefore, facilitate corticospinal excitability to a greater extent than passive observation instructions.

Puglisi et al. [17] explored this issue by measuring the amplitude of the H-reflex during the action observation of a rhythmical hand flexion-extension movement with different attentional manipulations. In one condition participants were directed explicitly to focus on the action, whilst in two other conditions participants observed the same action but were instructed to complete cognitive a task involving monitoring the activity of a light mounted onto the hand, rather than to focus on the action itself. Relative to the control condition, the amplitude of the H-reflex was increased in all three conditions, but the effect was significantly greater in the condition where attention was directed explicitly to the observed action. The authors interpreted this to indicate that the motor resonance response to action observation is more pronounced when attention is directed explicitly to the observed action.

In a similar experiment using TMS, D’Innocenzo et al. [18] examined the effect of directing participants’ visual attention to specific fixed locations of an observed thumb abduction-adduction movement. They reported that, relative to a free-viewing condition where visual attention was not constrained, corticospinal excitability was facilitated when participants directed their visual attention to a fixed location that would have provided the most biological motion across the fovea. Betti et al. [19] also demonstrated that corticospinal excitability is reduced when attention is drawn away from an observed action by the introduction of a flashing dot in a location contralateral to the observed action. Based on these findings, directing visual attention explicitly during action observation interventions appears to facilitate corticospinal excitability, and may accelerate motor (re)learning through observation [18]. Despite this emerging body of research indicating a link between visual attention and modulation of corticospinal excitability during action observation, further research is required. To date, only D’Innocenzo et al. [18] have recorded TMS and eye-tracking data simultaneously and so the direct relationship between visual attention and corticospinal excitability remains to be established. In particular, it is currently unknown whether certain eye-gaze metrics may predict modulation of corticospinal excitability during action observation. Furthermore, it is possible that directing visual attention to different aspects of on observed action, such as to specific body parts or objects involved, may differentially modulate corticospinal excitability, but this has not yet been tested.

This experiment aimed to determine the extent to which directing participants’ visual attention to different task-relevant features of an action, observed from a first-person visual perspective, modulated corticospinal excitability. It was hypothesised that: (i) corticospinal excitability would be facilitated during action observation and this facilitation would be specific to the muscles involved in the execution of the observed action, (ii) this facilitation would be greater when participants were instructed to direct their visual attention to specific task-relevant features of the observed action, compared to when they observed freely, and (iii) the number of fixations located on task-relevant features of the observed action would significantly predict the extent of corticospinal excitability during action observation.

## Material and methods

### Participants

Nineteen healthy volunteers (13 male, 6 female) aged 19–37 years (mean age 23.63 ± 4.59 years) participated in the experiment. Seventeen participants were right handed and two were left handed, as assessed by the Edinburgh Handedness Inventory [26]. All participants had normal or corrected-to-normal vision. Prior to participation, the TMS Adult Safety Screen [27] was used to identify participants who may have been predisposed to possible adverse effects of the stimulation. None of the sample were excluded from the experiment based on their questionnaire responses, and no participants reported experiencing adverse effects during the experiment. The Exercise and Sport Science Departmental Ethics Subcommittee at Manchester Metropolitan University granted ethical approval for this research, and written informed consent to take part in the experiment was obtained from all participants.

### Electromyography and transcranial magnetic stimulation procedure

Electromyography (EMG) was recorded throughout the experiment from the first dorsal interosseous (FDI) and abductor digiti minimi (ADM) muscles of the right hand, using a Delsys Bagnoli-4 EMG system and DE-2.1 bipolar single differential surface EMG electrodes (Delsys, Boston, MA, USA). Electrodes were placed over the mid-point of the belly of the muscle, with a reference electrode placed on the ulnar process of the right wrist. The EMG signal was recorded using Spike 2 (version 6.18) software with a sampling rate of 2 kHz, bandwidth of 20–450 kHz, 92 dB common mode rejection ratio and >10^15^ Ω input impedance, received by a Micro 1401–3 analogue-to-digital converter (Cambridge Electronic Design, Cambridge, UK).

Single-pulse TMS was delivered to the hand representation of the left motor cortex using a figure-of-eight shaped coil (two 70 mm diameter loops) connected to a Magstim 200^2^ magnetic stimulator (Magstim, Whitland, Dyfed, UK). The coil was orientated at a 45° angle to the central line between the nasion and inion landmarks of the cranium [28] and was held in place against the optimal scalp position (OSP) using a mechanical arm. The OSP was identified as the scalp location that produced motor evoked potentials (MEPs) of largest amplitude in the FDI and ADM muscles, using a stimulation intensity of 60% maximum stimulator output. This stimulation intensity is commonly used to identify the OSP in TMS action observation research [29, 30, 31, 32] as it produces MEPs of large amplitude in most individuals. Once identified, the OSP was marked on a tightly fitting polyester cap worn by participants and accurate coil placement was ensured throughout the experiment by checking the coil position frequently against this mark. Each participant’s resting motor threshold (RMT) was then determined by gradually reducing or increasing the stimulation intensity until the minimum intensity capable of producing MEPs in excess of 50 μV in 5 out of 10 trials was identified [33, 34]. RMT values ranged from 39–61% maximum stimulator output, and the stimulation intensity for the experiment was set at 110% RMT, based on Loporto et al.’s [30] recommendations.

### Eye-tracking procedure

Eye-movements were recorded throughout the experiment at a sampling rate of 25 Hz, using an Applied Science Laboratories Mobile Eye gaze registration system (Applied Science Laboratories, Bedford, MA, USA). The system uses lightweight spectacles fitted with two cameras to record eye movements in relation to the visual scene. A circular cursor, representing 1° of visual angle with a 4.5 mm lens, indicated the location of gaze in a video image of the scene (spatial accuracy of ±0.5° visual angle; 0.1° precision) and was recorded for subsequent analysis. Prior to commencing the experiment, the eye-tracker was calibrated using a nine-point calibration chart on the screen and this calibration was monitored throughout the experiment via a laptop situated directly behind the participant.

### Experimental procedure

Participants were seated on a chair with the head positioned comfortably between an adjustable head-and-chin rest and the TMS coil to minimise movement and maintain an accurate OSP. Participants’ forearms and hands rested in a pronated position on a black-painted table located in front of the participants and were obscured from their view by a black-painted wooden cover. A 32-inch Samsung flat-screen TV was positioned at eye-level in front of participants at a distance of 90 cm. The room was dimly illuminated and blackout curtains were drawn along either side of the table to eliminate any distracting visual stimuli in the participant’s peripheral field of vision.

**Fig 1:**
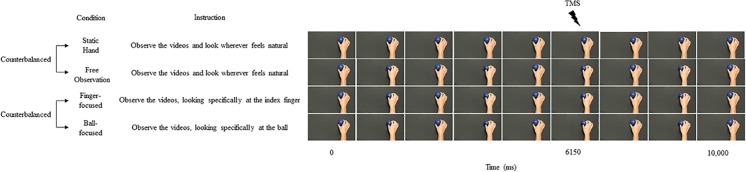
A representation of the four experimental conditions. Screenshots taken from the videos presented for each condition depict the ball either being held or pinched between the index finger and thumb.

Participants took part in four experimental conditions, termed: Static Hand, Free Observation, Finger-focused Observation and Ball-focused Observation (see Fig 1). In the static hand condition participants were shown videos of a still right hand holding a ball between the thumb and index finger and were instructed to “Observe the videos and look wherever feels natural”. In the free observation condition, participants were shown videos of a right hand pinching a ball between the thumb and index finger at a frequency of 0.4 Hz and were instructed to “Observe the videos and look wherever feels natural”. In the finger-focused condition, participants observed the same video used in the free observation condition, but were instructed to “Observe the videos, looking specifically at the index finger”. In the ball-focused condition, participants again observed the same video used in the free observation condition, but were instructed to “Observe the videos, looking specifically at the ball”. All videos were filmed from a first-person visual perspective with the forearm and hand positioned to the right of the screen, to be in line with the positioning of the participant’s own limb. A first-person visual perspective was used as action observation from this perspective has been shown to facilitate corticospinal excitability to a greater extent than action observation from third-person visual perspectives [35, 36]. The model used in the video had no distinguishing anatomical features and no jewellery was worn. The skin tone of the model’s arm and hand was similar to that of all participants. To ensure that the directed instructions did not influence participants’ viewing behaviour in the non-directed conditions, the static hand and free observation conditions were always presented as the first two conditions in a counterbalanced manner, followed by the counterbalanced presentation of finger-focused and ball-focused conditions. There were 25 trials of 10 s duration for each condition and one stimulation was delivered per trial. All videos were presented using DMDX video presentation software. A bespoke script, run through the Spike 2 software, was used to mark the onset of each video on the continuous EMG recording and then deliver the stimulation 6150 ms after the onset of the video. This time point corresponded to the point at which the FDI muscle of the model performing the action would have been at maximum contraction in the squeeze phase of the third pinch in each video [37]. Following each experimental condition participants were given a rest period of approximately two minutes.

### Data analysis

#### TMS data

As increased EMG activity at the time of stimulation may facilitate the amplitude of the subsequent MEP [38, 39], the amplitude of EMG activity 200 ms prior to each stimulation was measured in both muscles. Any trials in which a participant’s baseline EMG amplitude during this period was greater than 2.5 SD higher than that participant’s mean baseline EMG amplitude for that muscle were discarded from the analysis. This resulted in a mean of 2.84 (± 3.76) trials being discarded per participant from the FDI muscle and 2.79 (± 3.63) trials being discarded from the ADM muscle. In addition, in the finger-focused and ball-focused conditions, any trials in which a participant’s gaze was not located on the target to which it had been directed at the time of the TMS stimulation were removed from the data set. This resulted in a mean of 1.63 (± 1.46) trials being removed per participant from the finger-focused condition and 1.58 (± 2.04) trials being removed from the ball-focused condition. The peak-to-peak amplitude of MEPs in the remaining trials was then measured and averaged for each condition. The MEP amplitude data were then converted into z-scores [12, 25, 30, 40] and analysed using a 2 (muscle: FDI, ADM) x 4 (condition: static hand, free observation, finger-focused, ball-focused) repeated measures analysis of variance (ANOVA). Post-hoc pairwise comparisons with the Bonferroni adjustment were applied where necessary.

#### Eye-tracking data

**Fig 2:**
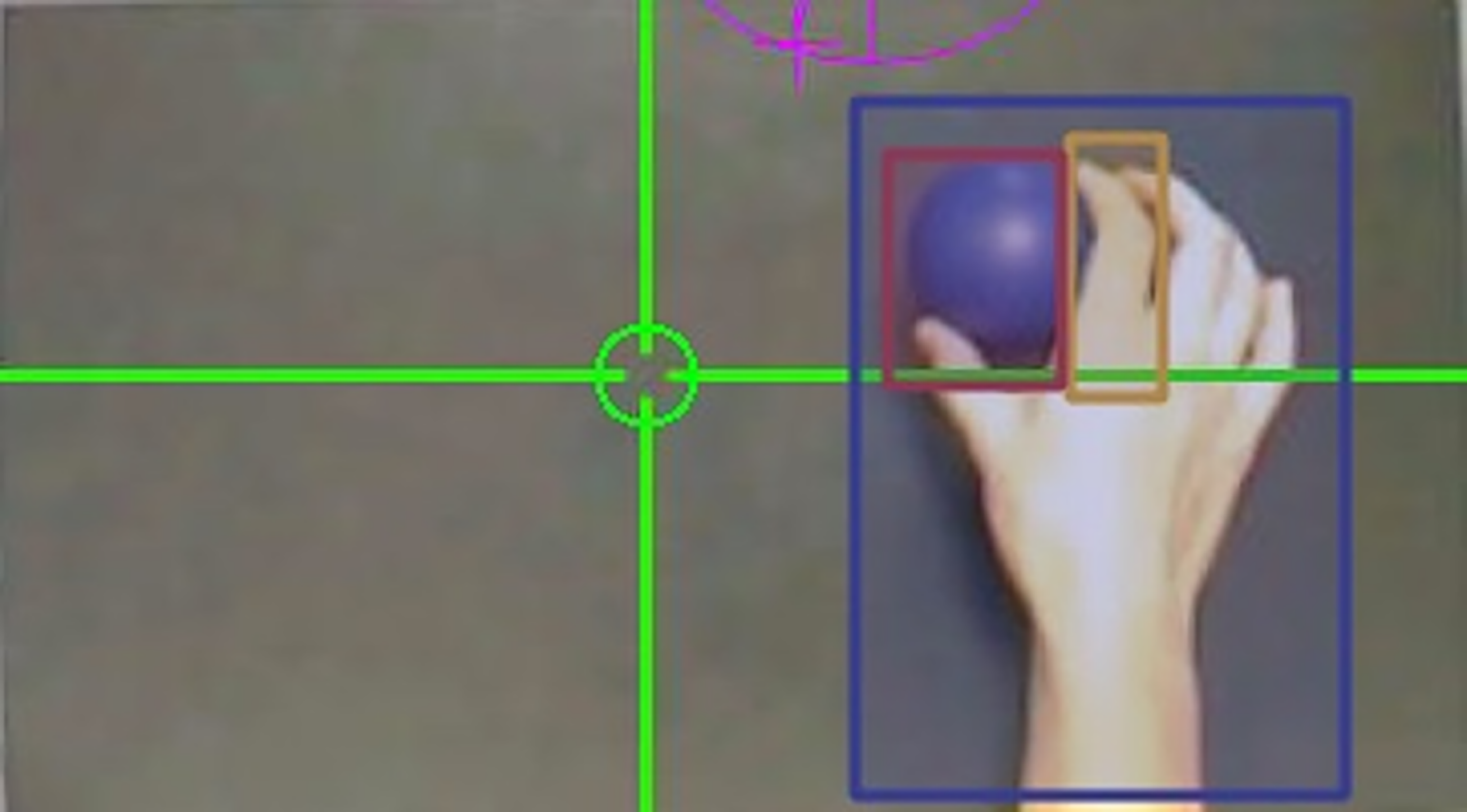
Areas of interest (AOIs) around the hand (blue box), index finger (orange box) and ball (red box) from which eye-tracking fixation data were extracted. A fourth area of interested, termed ‘other’ comprised any part of the visual display outside of the hand, index finger and ball AOIs.

Gaze data were analysed using ASL GazeMap software (Applied Science Laboratories, Bedford, MA). Each condition was split into 25 separate trials. Individual trials were then analysed by drawing three separate areas of interest (AOIs) around the hand, index finger and ball (see Fig 2). A fourth AOI termed ‘other’ comprised any part of the visual display outside of the hand, index finger and ball AOIs. AOIs were manipulated slightly (via frame-by-frame analysis) to account for the dynamic nature of some of the videos. This ensured that each AOI remained in position despite, for example, finger movement or the slight head movement of the participant [41]. GazeMap software then automatically calculated the number of fixations and the percentage of time spent fixating in each AOI. A fixation was defined as any gaze that remained stable (within 1 degree of visual angle) for a duration of over 100ms [42]. As the index finger and ball AOIs were included within the hand AOI, the number of fixations within these two AOIs were subtracted from the hand AOI fixation data. Gaze data were then analysed using two 4 (condition: static hand, free observation, finger-focused, ball-focused) x 4 (AOI: hand, finger, ball, other) ANOVAs. Significant interactions were then followed up with separate repeated measures ANOVAs for each condition followed by Bonferroni corrected pairwise comparisons. Finally, two separate linear regression analyses were carried out for each viewing condition (i.e., finger-focused or ball-focused) for each gaze variable (i.e., number of fixations and percentage fixation duration).

For all analyses, the alpha level for statistical significance was set at p ≤ .05 and effect sizes are reported as partial eta squared (η^2^_ρ_). Where Mauchley’s test indicated that the assumption of sphericity was violated the degrees of freedom were corrected using the Greenhouse-Geisser method. All statistical analyses were conducted using the IBM SPSS Statistics 21 software package.

## Results

### Post-hoc power analysis

A post hoc power analysis was conducted using G*power for the four conditions on data from the FDI muscle. The power analysis results between the static hand and free observation conditions were power = .89 (a = .05; b = .11); between the static hand and finger-focused conditions were power = .99 (a = .05; b = .01); and between the static hand and ball-focused condition were power = .99 (a = .05; b = .01). Between the free observation and finger-focused condition, the results were power = .76 (a = .05; b = .24) and between the free observation and ball-focused condition were power = .99 (a = .05; b = .01). These results indicate that the experiment had sufficient power to find the predicted significant differences.

### TMS findings

**Fig 3:**
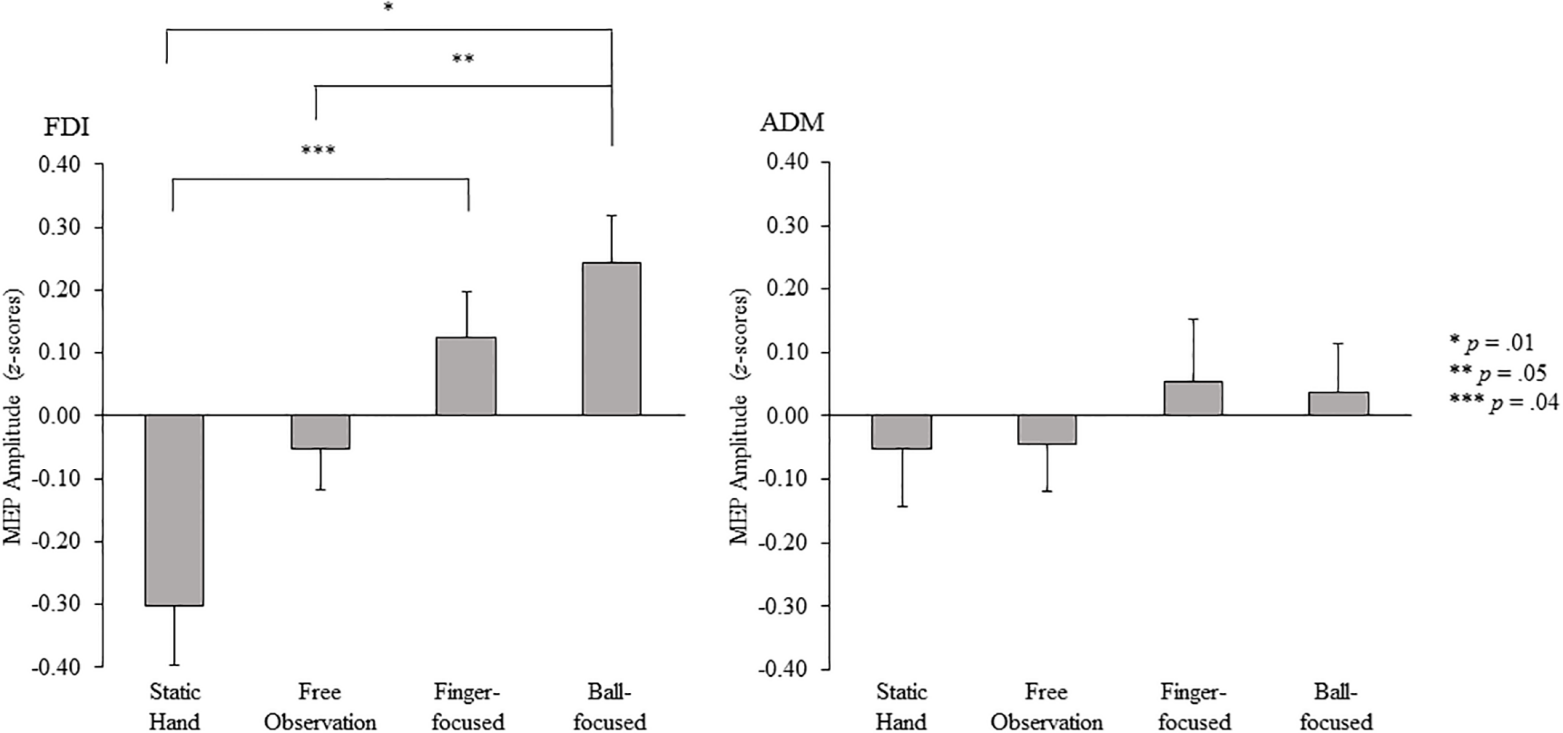
Mean MEP amplitudes from the FDI and ADM muscles, displayed as z-scores, for the static hand, free observation, finger-focused and ball-focused conditions. Positive z-score values indicate that the MEP amplitude in that condition was greater than the mean MEP amplitude in that muscle across all conditions. Negative z-score values indicate that the MEP amplitude in that condition was less than the mean MEP amplitude in that muscle across all conditions.

Raw MEP amplitude values obtained from the FDI and ADM muscles for each of the four experimental conditions can be found in Table 1. The repeated measures ANOVA conducted on the z-score MEP amplitude data showed a significant muscle x condition interaction effect, F_(3,54)_ = 3.17, p = .03, η^2^_ρ_ = .15 (see Fig 3). Pairwise comparisons with the Bonferroni adjustment indicated that in the FDI muscle, MEP amplitudes were significantly larger in the finger-focused (p = .04) and ball-focused (p = .01) conditions, compared to the static hand condition. In addition, MEPs in the ball-focused condition were also significantly larger than those obtained in the free observation condition (p = .05). There were no significant differences in MEP amplitude between any conditions in the ADM muscle.

**Table 1:** Mean raw MEP amplitude values (± SE) from the FDI and ADM muscles for the static hand, free observation, finger-focused and ball-focused conditions.

	Raw MEP Amplitude (μV)	
	FDI	ADM
Static Hand	1330.05 (± 201.59)	793.88 (± 119.61)
Free Observation	1673.29 (± 273.65)	809.59 (± 129.14)
Finger-focused Observation	1861.97 (± 267.28)	914.22 (± 186.71)
Ball-focused Observation	2073.15 (± 357.60)	897.93 (± 156.03)

### Eye-tracking findings

#### Number of fixations

**Fig 4:**
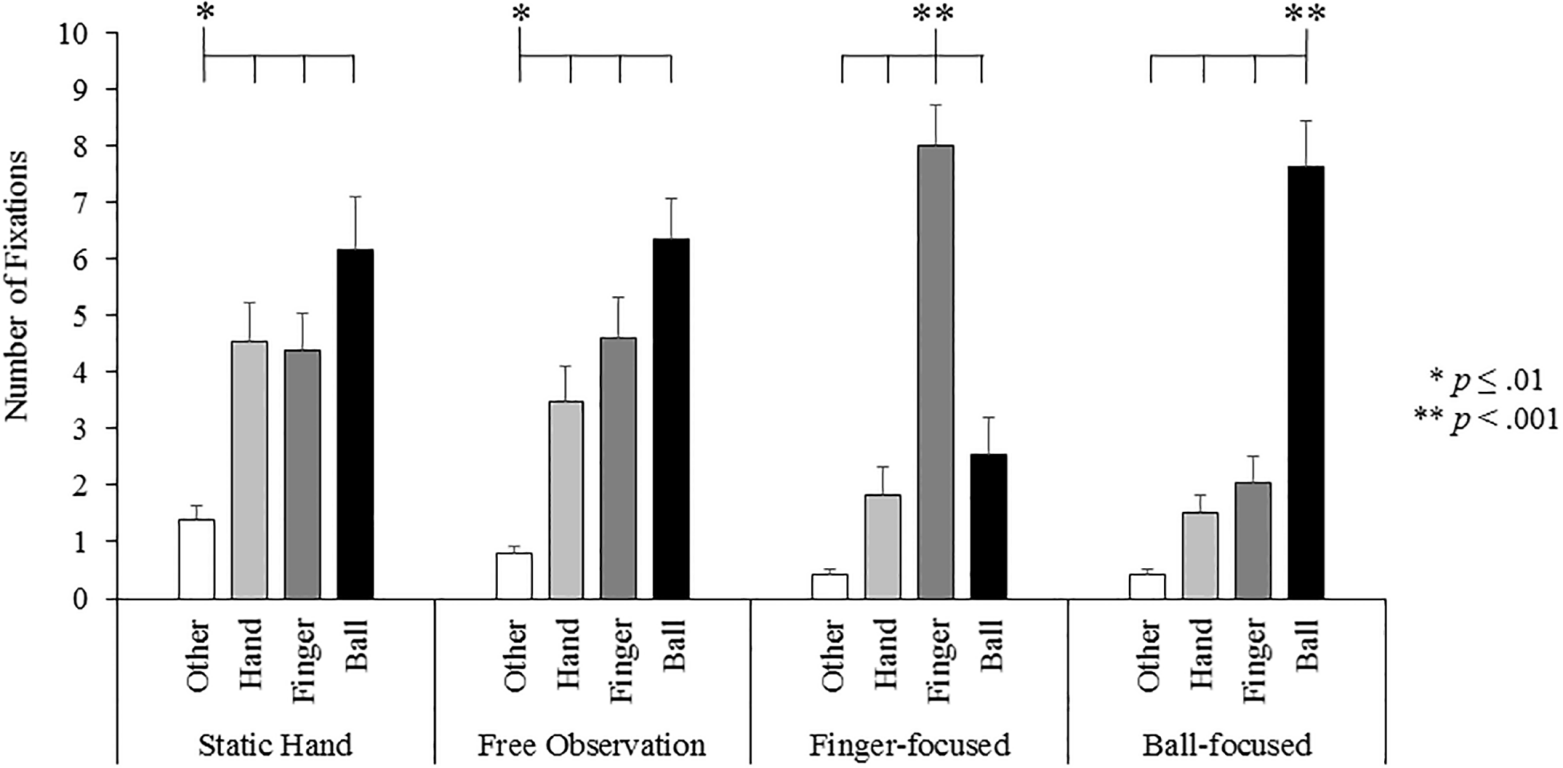
Total number of fixations made in each area of interest for the static hand, free observation, finger-focused and ball-focused conditions.

A significant condition x AOI interaction effect was found, F_(4.23,76.17)_ = 16.32, p < .001, η^2^_ρ_ = .48 (see Fig 4). Further ANOVAs across each condition revealed that in the static hand condition participants displayed significantly more, F_(2.12,38.20)_ = 7.75, p = .001, η^2^_ρ_ = .30, fixations on the hand (p < .001), ball (p = .001) and finger (p = .01) compared to the other areas of the display. Similarly, in the free observation condition participants displayed significantly more, F_(2.12,38.13)_ = 13.54, p < .001, η^2^_ρ_ = .43, fixations on hand (p = .002), ball (p < .001) and finger (p < .001) compared to other areas of the display. In the finger-focused condition, participants directed significantly more, F_(2.20,39.58)_ = 39.95, p < .001, η^2^_ρ_ = .69, fixations towards the finger compared to the hand (p < .001), ball (p < .001) and other (p < .001) areas of the display. In the ball-focused condition, participants directed significantly more, F_(1.68,30.24)_ = 40.94, p < .001, η^2^_ρ_ = .70, fixations towards the ball compared to the hand (p < .001), finger (p < .001) and other (p < .001) areas of the display.

#### Percentage fixation duration

**Fig 5:**
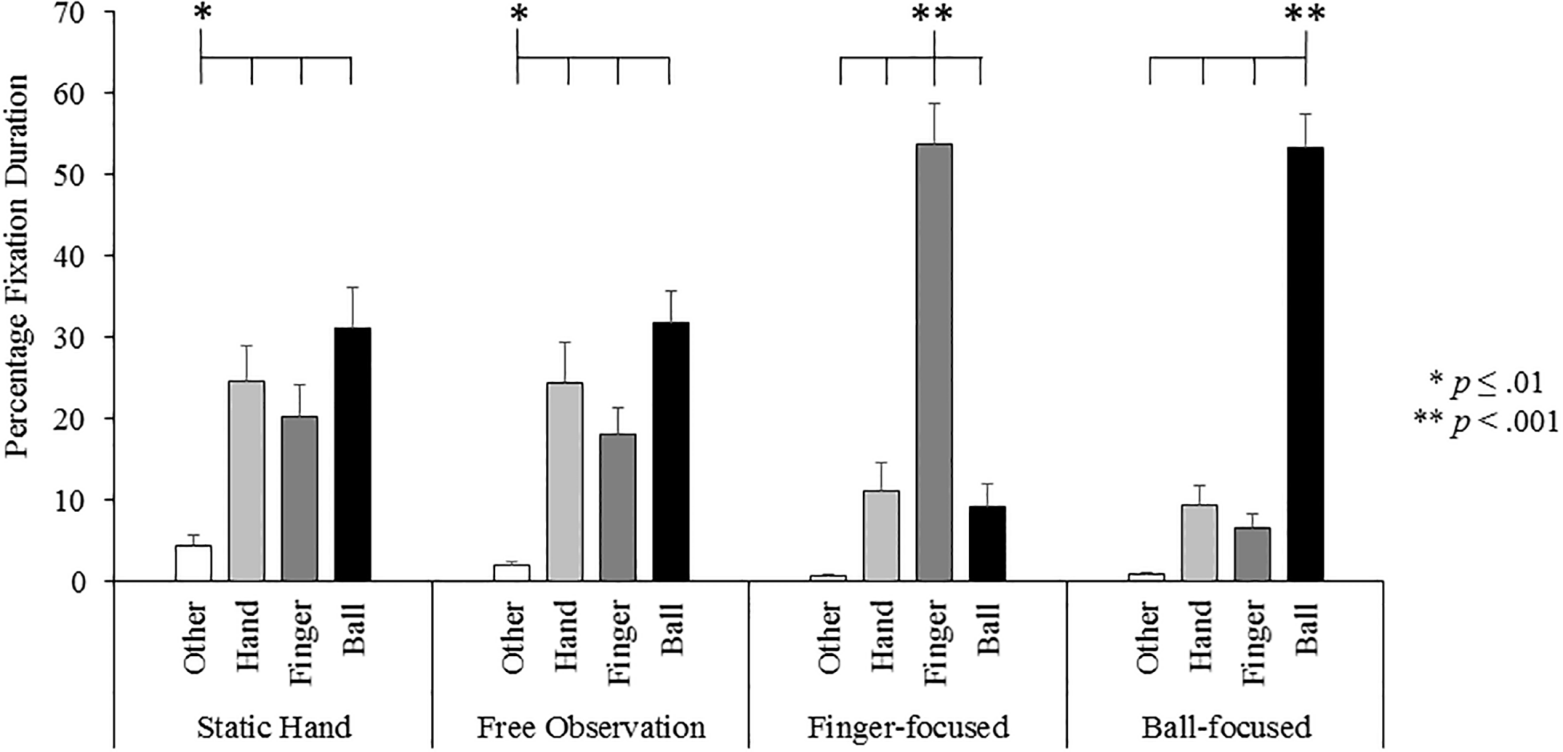
Percentage of time spent fixating in each area of interest for the static hand, free observation, finger-focused and ball-focused conditions.

A significant condition x AOI interaction effect was found, F_(3.99,71.96)_ = 25.60, p < .001, η^2^_ρ_ = .59 (see Fig 5). Further ANOVAs across each condition revealed that in the static hand condition participants spent significantly longer, F_(1.81,32.56)_ = 6.61, p = .005, η^2^_ρ_ = .27, fixating on the hand (p < .001), ball (p = .001) and finger (p = .014) compared to the other areas of the display. In the free observation condition participants spent significantly longer, F_(1.97,35.38)_ = 10.22, p < .001, η^2^_ρ_ = .36, fixating on hand (p = .001), ball (p < .001) and finger (p = .001) compared to other areas of the display. In the finger-focused condition, participants spent significantly longer, F_(1.58,28.38)_ = 38.39, p < .001, η^2^_ρ_ = .68, fixating on the finger compared to the hand (p < .001), ball (p < .001) and other (p < .001) areas of the display. In the ball-focused condition, participants spent significantly longer fixating, F_(1.56,28.13)_ = 75.75, p < .001, η^2^_ρ_ = .81, on the ball compared to the hand (p < .001), finger (p < .001) and other (p < .001) areas of the display.

### Regression findings

The number of fixations on the ball was the only significant predictor of MEP amplitude in the ball-focused condition (R^2^ = .30, b = .55, p = .02). The percentage duration spent fixating on the ball did not significantly predict MEP amplitude in this condition (R^2^ = .08, b = .27, p = .24). In the finger-focused condition, neither the number of fixations (R^2^ = .18, b = .42, p = .07) nor the percentage of fixation duration on the finger (R^2^ = .01, b = .11, p = .66) predicted MEP amplitude.

## Discussion

The primary aim of this experiment was to establish whether directing participants’ visual attention to task-relevant features of an observed ball pinch action would facilitate corticospinal excitability to a greater extent than free observation, where no specific viewing instructions were provided. Free observation of the ball pinch task did not facilitate corticospinal excitability in comparison to observation of the static hand. However, corticospinal excitability was facilitated when participants observed the same movement but were directed explicitly to focus their visual attention towards either the index finger or ball. Furthermore, when participants’ visual attention was directed towards the ball, but not the index finger, corticospinal excitability was facilitated to a greater extent than during free observation. The number of fixations on the ball in the ball-focused condition was also found to predict MEP amplitude, with more fixations on the ball predictive of a larger amplitude MEP response. Taken together, these results indicate that, for this type of action, instructing participants to direct their visual attention during action observation to the object with which the model interacts facilitates corticospinal excitability to a greater extent than free observation instructions. In addition, although not the main aim of this experiment, it is noteworthy that changes in corticospinal excitability during action observation were only found in the FDI muscle, but not in the ADM muscle. This finding is consistent with the well-established muscle-specific facilitation effect (see [14]) where facilitation of corticospinal excitability during action observation is specific to the muscles that would be involved in performing the observed action.

These results support the findings of the growing body of research which indicates that visual attention modulates corticospinal excitability during action observation [15, 16], and that directing visual attention towards specific features of the observed action can facilitate corticospinal excitability [17, 18]. The inclusion of eye-tracking measures in the current experiment, recorded simultaneously alongside TMS, extend the findings of this previous research. Donaldson et al. [16] reported that during free observation there was a significant positive correlation between the number of fixations on an effector-object interaction (a right hand gripping a spoon handle and using it to stir a cup of tea) and MEP amplitude in the observers’ right hand muscles. D’Innocenzo et al. [18] then demonstrated that directing visual attention explicitly to a fixed location which resulted in increased detection of biological motion across the fovea facilitated corticospinal excitability to a greater extent than free observation. The findings of the current experiment extend these results by providing the first evidence that, for the observation of a ball pinch action, directing visual attention explicitly to the ball, but not the index finger, predicts larger MEP responses in the muscle involved in the execution of the observed action. As such, directing visual attention to the object with which the model interacts during action observation may access the observer’s motor system to a greater extent than free observation instructions.

Activity in the putative human mirror neuron system is often assumed to be the mechanism by which corticospinal excitability is facilitated during action observation. Mirror neurons are active during both the execution and observation of movements and are proposed to exist in a network of brain regions which includes the premotor cortex [43]. Strong cortico-cortical connections are thought to link the premotor and motor cortices [44]. As such, the facilitation of corticospinal excitability following stimulation of the primary motor cortex during action observation is typically interpreted to reflect an increase in activity of neurons with mirror properties in the premotor cortex. The premotor cortex also contains canonical neurons, which become active in response to both the presentation of an object and when physically interacting with an object [45, 46]. The facilitation of corticospinal excitability in the ball-focused condition, relative to free observation, may, therefore, reflect increased activity in both mirror and canonical neurons within the premotor cortex when visual attention was directed to the ball. This seems plausible as when fixating on the ball, participants would have perceived information related to the object, but would also have perceived information regarding the movement of the index finger. This would have been particularly evident at the time points when the ball was pinched and the TMS was delivered. When visual attention was directed explicitly to the index finger, whilst there may have been increased activity in neurons with mirror properties, activity in canonical neurons of the premotor cortex may have been reduced as participants perceived less information about the object and its affordances, such as how it was mis-shapen by the pinch. This may explain why corticospinal excitability was not facilitated relative to free observation in the finger-focused condition.

In addition to increasing activity in both mirror and canonical neurons, another explanation for these findings is that directing participants’ visual attention to the ball may have allowed participants to infer more about the intention and the goal of the observed action than when they observed freely or directed their visual attention to the index finger. Although it is well-established that action observation facilitates corticospinal excitability [13, 14], there is evidence that observation of goal-directed actions results in an increased facilitation of corticospinal excitability than observation of actions without a clear goal. For example, Enticott et al. [47] reported that observation of a transitive grasping action, in which a hand interacted with an object, facilitated corticospinal excitability but no such effect was found for observation of a mimed intransitive grasping action that did not involve interaction with an object. In addition, Enticott et al. [47] reported that the MEP response in the transitive action observation condition was significantly greater than in the intransitive action observation conditions. In a related experiment, Donne et al. [48] reported that observation of meaningless actions such as thumb tapping did not facilitate corticospinal excitability in comparison to a control condition. However, observation of goal-directed actions (e.g., grasping a pen) or social actions (e.g., thumb wrestling) were found to facilitate corticospinal excitability. Although the finger- and ball-focused conditions in the current experiment both involved observation of the same goal-directed action, the fact that visual attention was focused almost exclusively on the goal of the action in the ball-focused condition may provide the explanation for why corticospinal excitability was facilitated to a greater extent in this condition, compared to when participants observed freely.

This explanation is supported by the findings of Cattaneo et al. [49]. In this experiment, MEPs were recorded from the opponens pollicis (OP) muscle whilst participants observed both normal pliers (i.e., opened by extension of the thumb and index finger) or reverse pliers (i.e., opened by flexion of the thumb and index finger) either being opened and closed aimlessly in a ‘no-goal’ condition, or used to pick up an object in a ‘goal-directed’ condition. When no goal was evident, facilitation of MEP amplitude reflected the involvement of the OP muscle in the observed action, regardless of the type of pliers used. In the goal-directed conditions, however, facilitation of MEP amplitude in the OP muscle was instead facilitated by goal achievement irrespective of the involvement of the OP muscle in the observed action. The findings have been interpreted as an indication that, when observing goal-directed tasks, the motor system of the observer prioritises the end-goal of the observed movement, regardless of the kinematics of the observed action [49, 50, 51].

Another possible explanation for the increased MEP response relative to free observation in the ball-focused condition, but not the finger-focused condition, may relate to the way in which participants were able to perceive information about the force required to perform the observed movement. Alaerts et al. [52] conducted two experiments in which participants observed a model reaching, grasping and lifting objects of different weights. The force requirements of the observed action were found to modulate corticospinal excitability, with larger increases in corticospinal excitability reported when participants observed the lifting of the heavier, compared to lighter, weighted objects. Similar results have been reported in several other TMS experiments (e.g., [53]). In the current experiment, it is possible that participants were able to perceive more about the force requirements involved in the observed action when their visual attention was directed to the ball and they focused on how it was mis-shapen as it was manipulated by the model. In contrast, when participants’ visual attention was directed towards the index finger it is possible that they were able to perceive less about the force required to perform the observed action as they were only focusing on tracking the finger move back and forth and not on how the object was manipulated.

Although these findings extend the literature regarding the influence of visual attention on the modulation of corticospinal excitability during action observation, aspects of the current findings differ to previous research. D’Innocenzo et al. [18] reported that for the observation of an intransitive thumb abduction-adduction movement, directing visual attention to a fixed point that maximised perception of biological motion across the fovea facilitated corticospinal excitability, compared to free observation. The finger-focused condition in the current experiment would have provided the most biological motion information across the fovea, but this condition did not produce a significant facilitation of corticospinal excitability relative to the free observation condition. A facilitation effect relative to free observation was, however, found for the ball-focused condition. The optimal location to which visual attention should be directed during action observation, therefore, remains to be established. Although speculative at this stage, it is possible that for the observation of intransitive actions corticospinal excitability may be best facilitated by directing the observers’ visual attention in a manner that encourages greatest perception of biological motion [18]. For the observation of transitive actions, however, the results of the current study indicate that directing visual attention to the object with which the model interacts, rather than the effector itself, may best facilitate corticospinal excitability. Future research should seek to confirm this suggestion by comparing the effect of directing visual attention to different components of both transitive and intransitive actions within a single experiment.

The findings reported in this experiment have important implications for motor (re)learning settings. Action observation has been recommended as an effective intervention for improving aspects of motor function, and there is a growing body of literature justifying the efficacy of the technique (see [11]). The findings from action observation experiments using TMS have informed the design and delivery of such interventions. Specifically, there is an implicit assumption that action observation conditions that produce the largest facilitation in corticospinal excitability may offer a more effective strategy for motor (re)learning [54]. A ball pinch action was used in this experiment because it is a similar action to what stroke survivors may practice during rehabilitation what attempting to regain motor function in the affected limb. Action observation interventions in such settings typically instruct observers to observe freely or observe with the intent to imitate the action [11], with more recent research also suggesting combining observation with concurrent and congruent imagery [55, 56]. Precise instructions regarding where observers should direct their visual attention whilst engaging in action observation interventions are rarely provided or reported. The findings reported in this experiment indicate that directing a participant’s visual attention explicitly to the object with which the model interacts accesses the observer’s motor system to a greater extent than during free observation, when the observer’s visual attention is not directed explicitly. Furthermore, the results indicate that directing the observer to fixate specifically on the object in the observed movement, rather than the movement itself, results in stronger activation of the observer’s motor system and may, therefore, offer the more effective viewing instruction for motor (re)learning. Future research should seek to investigate the efficacy of directing visual attention to specific features of the observed action during action observation interventions for motor (re)learning in situations where an individual’s motor function has been compromised, for example, following stroke or in patients with Parkinson’s Disease.
